# Progress towards the child mortality millennium development goal in urban sub-Saharan Africa: the dynamics of population growth, immunization, and access to clean water

**DOI:** 10.1186/1471-2458-7-218

**Published:** 2007-08-28

**Authors:** Jean-Christophe Fotso, Alex Chika Ezeh, Nyovani Janet Madise, James Ciera

**Affiliations:** 1 African Population and Health Research Center (APHRC), Nairobi, Kenya, Africa; 2 School of Social Sciences, University of Southampton, Southampton, UK; 3 Department of Statistics, University of Padova, Italy

## Abstract

**Background:**

Improvements in child survival have been very poor in sub-Saharan Africa (SSA). Since the 1990s, declines in child mortality have reversed in many countries in the region, while in others, they have either slowed or stalled, making it improbable that the target of reducing child mortality by two thirds by 2015 will be reached. This paper highlights the implications of urban population growth and access to health and social services on progress in achieving MDG 4. Specifically, it examines trends in childhood mortality in SSA in relation to urban population growth, vaccination coverage and access to safe drinking water.

**Methods:**

Correlation methods are used to analyze national-level data from the Demographic and Health Surveys and from the United Nations. The analysis is complemented by case studies on intra-urban health differences in Kenya and Zambia.

**Results:**

Only five of the 22 countries included in the study have recorded declines in urban child mortality that are in line with the MDG target of about 4% per year; five others have recorded an increase; and the 12 remaining countries witnessed only minimal decline. More rapid rate of urban population growth is associated with negative trend in access to safe drinking water and in vaccination coverage, and ultimately to increasing or timid declines in child mortality. There is evidence of intra-urban disparities in child health in some countries like Kenya and Zambia.

**Conclusion:**

Failing to appropriately target the growing sub-group of the urban poor and improve their living conditions and health status – which is an MDG target itself – may result in lack of improvement on national indicators of health. Sustained expansion of potable water supplies and vaccination coverage among the disadvantaged urban dwellers should be given priority in the efforts to achieve the child mortality MDG in SSA.

## Background

Improvements in child survival have been very poor in sub-Saharan Africa (SSA). Since the 1990s, declines in child mortality have reversed in many countries in the region, while in others they have either slowed or stalled, making it improbable that the target of reducing child mortality by two thirds by 2015 will be reached by the majority of the countries in the region. Under-five mortality rate (U5MR) in SSA varied from 185 (per 1,000 live births) in 1990 to 172 in 2003 [1,2]. This corresponds to an overall decline of about 7%, or nearly 0.5% on an annual basis, while the MDG targets an average reduction of 4.3% per year. If the region had been on track to meeting the MDG on child mortality, U5MR would be around 105 in 2003. At current trends, mortality rate in children younger than 5 years will decline by less than 15% by 2015 from the 1990 base year, compared to the expected goal of 66.7%.

It is estimated that more than 10 million children under the age of 5 years die each year, with about 90% of these deaths occurring in just 42 countries, 36 of which are in SSA [3]. Numerous studies on infant and under-five mortality in developing countries indicate that most of these deaths are from preventable causes – such as diarrhea, pneumonia, measles, malaria, HIV and AIDS, and the underlying malnutrition – and suggest that the goal of reducing childhood mortality by two-thirds by 2015 could be achieved if few known and effective child survival interventions could reach population groups that need them most [3-5]. These include immunization, safe water and sanitation, micronutrient supplementation, nutrition counseling, and in malaria-prone areas, insecticide-treated bed nets [6]. The task of scaling up child-health interventions to full coverage in countries with the highest mortality is within reach, and resources should be mobilized to match governments' and development partners' commitments with action [7].

### Why focus on urban sub-Saharan Africa?

In sub-Saharan Africa, the 1980s was largely dominated by the protracted economic recession that affected most developing countries, with negative effects on food security and various aspects of human development [8]. Countries were required by international lending institutions to implement structural adjustment programs meant to stabilize their economies. Real wages were reduced together with the provision of public social services; unemployment increased; and public support schemes in favour of agriculture and rural development were downscaled or abandoned [8,9]. This process resulted in increased migration flows from rural to urban areas, generally composed of disadvantaged families, who were likely to have a harder time coping in urban areas than in rural areas [10]. These trends were more pronounced in SSA than in other parts of the developing world. Between 1980 and 2000, the region's urban population grew by about 4.7% per year, compared to 3.5% for the developing countries as a whole [11], while at the same time, per capita gross domestic product (GDP) dropped by 0.8% per annum [12], and food production index per capita increased by only 0.2% per year [13].

As a result of this rapid urbanization in the context of poor economic performance and poor governance, a rapidly increasing proportion of urban dwellers are living below the poverty line in overcrowded slums and shantytowns. These slums are generally characterized by poor environmental and sanitation conditions, poor access to basic amenities and social and health services, and poor livelihood opportunities, which in turn worsen the residents' susceptibility to various health problems [14,15].

While national trends in child mortality are generally worrying, more worrying is the emerging body of knowledge suggesting that the worsening child health outcomes in SSA may be accentuated among the urban poor [16-19]. Not only are disparities widening between the poor and non-poor in urban areas, in some countries current levels of child indicators among the poor in urban areas exceed the levels for all other sub-groups including rural residents [15]. A number of studies have also suggested that the rural/urban ill-health and mortality gaps in SSA have narrowed in recent years, mainly as a result of stalling and even upturn in urban trends, as urban economic and environmental conditions have sharply deteriorated in rapidly growing cities [20,21].

**Figure 1:**
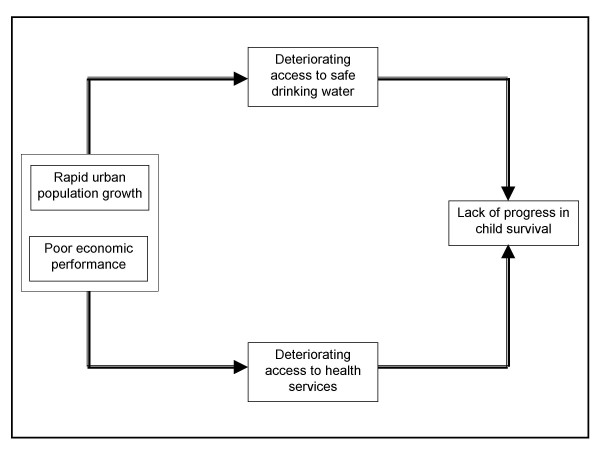
Urban population growth, access to water and to health services, and child mortality.

This study seeks to contribute to the growing evidence base on how far developing countries in general, and sub-Saharan African countries in particular, are progressing in reducing the persistent high child mortality rates. Though the MDGs have been set and are typically assessed at the national level, we argue that focus on urban areas of SSA provides a useful starting point for stocktaking of factors likely to get the region on track to meeting the child mortality target. More specifically, the goal of the paper is to assess the inter-relationships between urban population growth, trends in access to safe drinking water and in vaccination coverage, and trends in child mortality in urban areas of SSA. Despite the amount of work on child morbidity and mortality in developing countries [22-24], very few have focused on urban areas within a cross-country perspective. The paper is premised on a conceptual hypothesis which suggests that rapid urbanization amidst declining or stagnating economies is associated with poor access to safe drinking water and low health utilization, which in turn, lead to poorer or lack of progress in child survival in urban areas. This hypothesis is illustrated in Figure 1.

The importance of access to safe drinking water on child health, especially in urban areas, has been well documented as diarrhea remains the major cause of deaths among children under five [3,5,25]. In developing countries, migration streams to urban areas that have been the main fuelling factor of population growth in cities, strain existing water infrastructure. In this context, underprivileged urban populations often pay exorbitant prices for clean – and sometimes unclean – water, while services to wealthier groups are heavily subsidized [26]. Child immunization is another key factor influencing child survival in developing countries and has been considered as the most cost-effective health intervention [27]. Vaccine-preventable diseases remain major causes of morbidity and mortality in Africa. Reductions in vaccine-preventable diseases have been recorded following the introduction of appropriate vaccines for routine use in infants [3,28]. Child survival is also influenced by the HIV epidemic through several mechanisms including mother-to-child transmission and breast-feeding [29], and evidence from various African countries suggests that pediatric AIDS has become one of the leading factors of mortality [30]. However, the fact that child mortality declined steadily in some of the countries heavily hit by HIV/AIDS such as Malawi, and went down only minimally in some of the countries with low HIV prevalence like Senegal, suggests that AIDS may not be the only problem.

This study shows that more rapid rates of urban population growth are associated with negative trends in access to safe drinking water and in vaccination coverage, and ultimately to increasing or timid declines in child mortality. There is evidence of intra-urban disparities in child health in countries like Kenya and Zambia.

## Methods

This study uses data from the Demographic and Health Surveys (DHS) from 22 sub-Saharan African countries with two or more surveys carried out between the 1990s and the 2000s. DHS surveys provide detailed health information on women aged 15–49 years, on children born in the three or five years preceding the survey date, and on relevant household characteristics including the type of drinking water source. Data on population growth are from the Population Division of the United Nations. Univariate and correlation analysis are carried out on the following variables:

1. Annual urban population growth between 1980 and 2000.

2. Annual percentage change over time in urban under-five mortality rate. Rates for the ten-year period preceding the survey are used for the computation.

3. Annual percentage change over time in the proportion of urban households with access to clean water.

4. Annual percentage change over time in the proportion of children 12–23 months who are fully vaccinated. A child is fully vaccinated if he/she has received BCG, measles, and three doses of DPT and polio (excluding polio 0).

Table 1 shows the qualifying countries, the survey periods, and the variables presented above. The results of the macro-level correlation analysis are complemented by case studies using data from Kenya and Zambia. For Kenya, supplementary data are used from the Nairobi Cross-sectional Slum Survey (NCSS) carried out by the African Population and Health Research Center (APHRC) in 2000. The survey covered a representative sample of all slum settlements in Nairobi city. The study was designed to provide comparable data to the 1998 and 2003 Kenya Demographic and Health Surveys, with the aim of determining the magnitude of intra-urban health inequalities in Kenya. Based on census enumeration areas used in the 1999 Kenya National Census, a representative cross-sectional sample of households in all slum clusters of Nairobi was designed. Like in the DHS, enumeration areas (EAs) were selected at the first stage of sampling, while households were selected from sampled EAs at the second stage. In total, the NCSS administered interviews to 4,564 households and 3,256 women of reproductive age (15–49) [15]. The data, in conjunction with the Kenya DHSs, allow for comparative analyses of social, health and reproductive health indicators between respondents residing in the slums of Nairobi, and residents in other urban areas and rural Kenya.

**Table 1:** Trends in under-five mortality, population growth, access to clean water, and child vaccination

		First Survey Year^**a**^	Last Survey Year^**a**^	Under-five mortality rate^**b**^	Urban population growth^**c**^	Access to safe drinking water^**d**^	Full vaccination^**e**^
1	Benin	1996	2001	-2.3	5.6	2.8	2.2
2	Burkina Faso	1992/93	2003	-0.8	6.3	2.8	-0.5
3	Cameroon	1991	2004	-0.1	5.0	0.1	0.6
4	Chad	1996/97	2004	-0.8	4.1	8.1	0.3
5	Côte d'Ivoire	1994	1998/99	0.9	4.7	0.3	5.7
6	Eritrea	1995	2002	-5.6	3.6	1.7	1.6
7	Ghana	1993	2003	0.3	4.6	-0.5	0.6
8	Kenya	1993	2003	2.2	7.4	-2.0	-4.1
9	Madagascar	1992	2003/04	-5.6	4.7	-1.5	1.6
10	Malawi	1992	2004	-4.6	5.8	-0.8	-1.7
11	Mali	1995/96	2001	-0.6	5.2	3.3	-0.6
12	Mozambique	1997	2003	-0.8	6.7	-2.7	-0.8
13	Namibia	1992	2000	-6.7	4.9	0.2	1.9
14	Niger	1992	1998	-2.7	5.8	0.7	0.2
15	Nigeria	1990	2003	1.2	5.3	-4.9	-5.4
16	Rwanda	1992	2000	-1.2	7.8	1.1	-2.3
17	Senegal	1992/93	1997	-2.9	4.3	0.1	3.3
18	Tanzania	1992	2004	-3.2	7.2	-1.2	-0.2
19	Togo	1988	1998	-2.6	5.3	0.8	1.5
20	Uganda	1995	2000/01	-5.0	5.0	6.6	-5.1
21	Zambia	1992	2001/02	-0.8	2.2	-0.9	0.3
22	Zimbabwe	1994	1999	1.8	4.9	0.2	-5.0

^**a**^First and last demographic and health surveys (DHS) carried out between the 1990s and the 2000s.

^**b**^Annual variation of urban under-five mortality rate. Source: DHS.

^**c**^Annual growth of urban population (1980–2000). Source: Population Division, United Nations.

^**d**^Annual variation of the percentage of urban households with access to safe drinking water. Source: DHS.

^**e**^Annual variation of the percentage of urban children aged 12–23 months who are fully immunized. Source: DHS.

We use data from the NCSS to identify households that were probably located in slum areas in the Kenyan and Zambian DHSs. From the NCSS, only 7 percent of households in slums had own flush toilets. Using the absence of own flush toilet as an indicator of slums in both Kenya and Zambia, we were able to obtain intra-urban differences. The case studies contrast infant mortality rates for the five-year period preceding the survey between slum and non-slum sub-groups. There were problems with computing U5MR for some years because of small numbers of observations in some sub-groups. We also examine access to piped water and child immunization over time among slum and non-slum children in Kenya and Zambia.

The study reported in the paper did not require any ethical approval, as it mainly used secondary analysis of publicly available data from the well-known Demographic and Health Surveys conducted in most developing countries since the mid-1980s.

## Results

### Univariate results

As can be seen from Table 1, five of the 22 countries (Namibia, Eritrea, Madagascar, Uganda and Malawi) recorded declines in urban child mortality in line with the MDG target of about 4% per annum, while at the other end, Kenya, Zimbabwe and Nigeria witnessed a sharp increase. Between these two extremes, six countries (Tanzania, Senegal, Niger, Togo, Benin and Rwanda) had a slow decline in mortality ranging from 1% to 3.2% per annum; and in eight others (Zambia, Burkina Faso, Chad, Mozambique, Mali, Cameroon, Ghana and Côte d'Ivoire), child mortality almost remained unchanged, with annual change ranging from -0.9% to +0.9% per year.

Between 1980 and 2000, the lowest urban population growth was noted in Zambia (2.2% per year), and Eritrea, Chad and Senegal (between 3.6% and 4.5%), whilst five countries (Burkina Faso, Mozambique, Tanzania, Kenya and Rwanda) had annual urban population growth between 6.3% and 7.8%. With regard to access to safe drinking water, nine countries achieved improvement in access to clean water of between 0.7% (Niger) and 8.1% (Chad); seven others recorded a decline ranging from 4.9% (Nigeria) to 0.8% (Malawi); while the six remaining countries witnessed almost no change. Six countries witnessed a decline in the coverage of full vaccination ranging from 5.4% (Nigeria) to 1.7% (Malawi). At the other end of the scale, six others recorded an increase of between 1.6% (Madagascar) and 5.7% (Côte d'Ivoire).

### Correlation analysis

**Figure 2:**
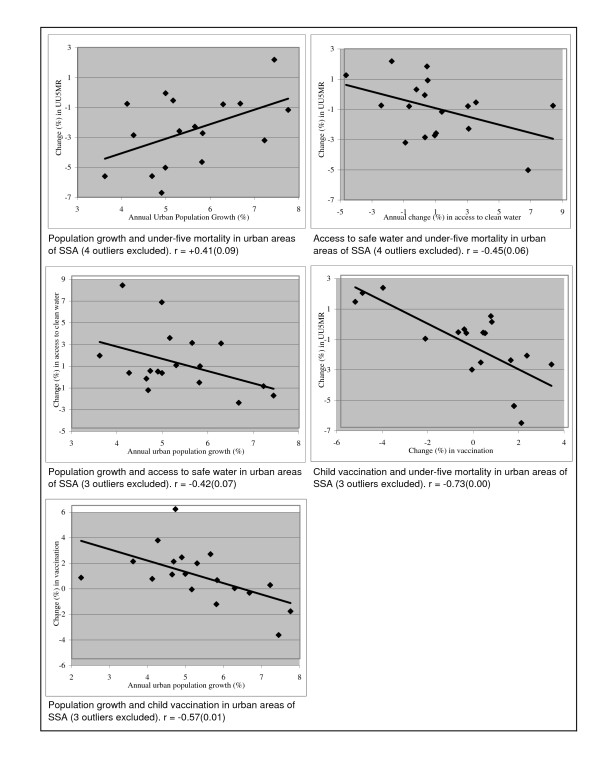
Trends in urban population growth, access to safe drinking water, vaccination, and under-five mortality.

Figure 2 displays the inter-relationships between trends in urban under-five mortality, urban population growth, and trends in access to safe drinking water and in vaccination coverage. The upper left graph shows the association between urban population growth and change over time in urban under-five mortality. As expected, it indicates that countries with more rapid rates of urban population growth tended to experience worsening trends or timid declines in urban child mortality. The correlation coefficient (+0.41) is statistically significant at the level of 0.10. Next, we investigate the extent to which access to clean water plays a role in the above association. As can be seen from the upper right graph of Figure 2, change over time in access to water is associated with trends in child mortality (correlation of -0.45; p value of 0.06). Countries with greater improvement in access to safe drinking water among the urban dwellers were likely to witness higher declines in under-five mortality. Moreover, the middle left graph indicates that higher rate of urban population growth is associated with deteriorating access to safe drinking water over time (correlation of -0.42; p value of 0.07).

Finally, the role of access to health services – proxied by child full vaccination – in the association between urban population growth and trend in child mortality is examined. The middle right graph of Figure 2 shows a very strong negative association between trends in child vaccination and change in under-five mortality (correlation of -0.73, p value of 0.00). It indicates that countries with greater improvement in child vaccination coverage consistently displayed more rapid declines in urban child mortality. Further, the bottom left graph indicates that higher rate of urban population growth is associated with decline or minimal increase in urban child vaccination coverage (correlation of -0.57, p value of 0.01). Overall, the inter-relationships between urban population growth, trend in access to clean water and health services, and change in under-five mortality appear to be consistent with our conceptual framework.

### Case studies of Kenya and Zambia

#### Kenya

Kenya typifies the current urban population boom and associated urban health and poverty problems. Between 1980 and 2000, its urban population increased at an annual rate of 7.2%, whilst per capita GDP dropped annually by about 0.1%. According to the 2003 DHS, about one quarter of Kenyans were living in urban areas. The Welfare Monitoring Survey shows that while absolute poverty increased from 48 to 53 percent in rural areas of Kenya between 1992 and 1997, it almost doubled from 27 to 50 percent over the same period in Nairobi City [31]. Nairobi has indeed grown into a poverty hub with more than half of its population estimated to be residing in slum settlements [32].

**Figure 3:**
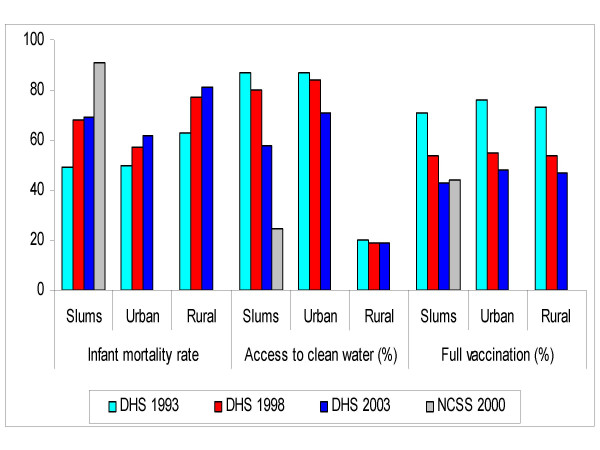
Infant mortality, access to safe water and full vaccination in Kenya.

As can be seen in Figure 3, infant mortality in Kenya has been increasing since the early 1990s. This increase in childhood mortality was observed in both rural and urban areas but was generally faster in slums than in rural areas. Between 1993 and 1998, the increase in infant mortality was about 39% in slums, compared with 14% and 22% in urban Kenya and rural areas, respectively. Using data from the NCSS of 2000, which was representative of slums of Nairobi, we see that slum children exhibit much higher infant mortality (91) than the average urban child, and more importantly, than those in rural areas of Kenya (81). Figure 3 also shows that access to clean water has been deteriorating in urban slums between 1993 and 2003. Slightly over half of households in slums had access to piped water in the 2003 DHS compared with 87 percent in the early 1990s. This represents a drop of about 33 percent, compared with 18 percent in urban Kenya and 5 percent in rural areas. The much lower figure reported from the NCSS data (24 percent) is due to the fact that water bought from street vendors were not classified as safe. Similarly, the proportion of urban children who were fully immunized dropped markedly from 76 percent in 1993 to 48 percent ten years later. Within slums, immunization rates were lower and dropped from 71 to 43 percent.

The disadvantage of the poor is not limited to child health. Detailed analysis of DHS data (results not shown) further indicate that in some countries like Kenya, while the fertility of the richest 20% has declined by more than 1.5 children in the last decade, that of the poorest 20% has either remained unchanged or has increased by more than one child in some countries. The poor have almost three times more children than the rich, they are also three times less likely to use contraceptives, and three times more likely to have unmet need for family planning. These results suggest that the high fertility of the poor may be largely unplanned or unwanted. With growing poverty and growing poor-rich fertility gap, greater proportions of children are increasingly born to poorer families [17]. This has implications for future population growth and the attainment of the health MDGs.

#### Zambia

Zambia is another interesting case study, exhibiting higher infant mortality than Kenya overall, but showing a mix of increasing and declining trends in the last few decades. Of particular focus to this paper are the intra-urban mortality differentials in childhood mortality and access to services and healthcare. The growth of urban centers in Zambia can be traced to the late 1950s where urban growth rates of more than 8 percent were recorded when copper mining was at its peak. Much of this growth was rural-urban migration as well as international migration. From mid 1970s, copper prices started to fall, thus affecting the economy and the provision of essential services. According to the United Nations Population Division indicators, the urban population growth rate was less than three percent between the early 1990s and mid 2000s. Despite the slow down in urban growth rate, about 40% of the Zambians reside in urban areas, which is high for the region. Between the 1960s and 1970s, infant mortality in Zambia declined from about 141 to 90 deaths per 1000 live births, but a reversal of this trend was noticed in the mid 1990s, with infant mortality rising to about 100 deaths per 1000 live births. Evidence of worsening child health among the urban poor was reported by Madise *et al*. who analyzed the 1992 and 1996 Zambian DHS data to identify changes in socioeconomic and demographic determinants of infant mortality [23]. They found that in the mid 1990s, children of the urban poor had 46 percent higher probability of dying in infancy than the poorest rural children. They also reported reversal in household socioeconomic status between the two surveys and lower utilization of health care.

**Figure 4:**
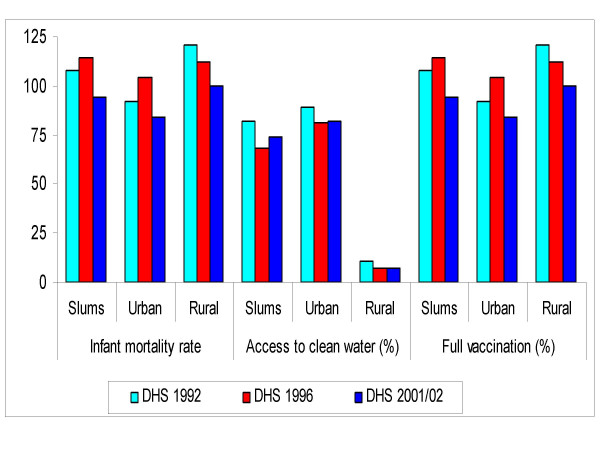
Infant mortality, access to safe water and full vaccination in Zambia.

Figure 4 lends further evidence of deteriorating child health among urban inhabitants between the early and mid 1990s. The pattern shows an increase in infant mortality in slums of about 7 percent compared with a reduction of 3 percent in rural areas between the 1992 and 1996 DHS surveys. While mortality in urban areas increased in the 1990s, the rural areas experienced sustained declined between the early 1990s and early 2000s. Access to piped water in slum areas declined between the early and mid 1990s, lending support to the poor provision of social services during the economic slump of the country. In 1992, 82 percent of households in the slums of Zambia had access to piped water, but this percentage dropped to 68 in the 1996 survey. The general decline in vaccination coverage between 1992 and 2001 was more pronounced in the slums (-13%) than in urban Zambia as a whole (-9%).

## Discussion

Declines in child mortality have been very poor in sub-Saharan African since the early 1990s, making it difficult for the region to meet – or at least get closer to – the MDG on child mortality. This paper has provided some degree of evidence on the linkages between urban population growth, change over time in access to safe drinking water and health services, and trends in child mortality. It has focused on urban areas of SSA – where health outcomes are generally perceived to be better and services more accessible – to highlight an issue that seems to be neglected: high rates of urban population growth will greatly undermine efforts to improve the wellbeing and reduce poverty, which is the underlying goal of the MDGs.

Our results show that only five of the 22 countries included in the study have recorded declines in urban child mortality that are in line with the MDG target of about 4% per year; five others have recorded an increase; and the remaining 12 countries have witnessed only minimal decline. The study has shown that more rapid rate of urban population growth is associated with increasing or timid declines in urban child mortality. Consistent with our initial hypothesis, poor access to safe drinking water and to health services are associated with periods of deteriorating child health. Higher urban population growth is associated with negative trends in access to safe drinking water, while at the same time, countries with greater improvement in access to safe drinking water among urban dwellers tended to experience higher declines in urban childhood mortality. Similarly, higher rate of urban population growth is associated with decline or minimal increase in child vaccination coverage, and countries with greater improvement in child vaccination coverage consistently displayed more rapid declines in urban child mortality.

Our findings concur with those of other studies that have shown the health effects of inadequate water supply and poor sanitation. It is estimated that almost half the people in the developing world have one of the main diseases related to inadequate water supply and sanitation, and that about 90% of diarrheal disease – the second leading cause of death among children under five years of age – is attributed to unsafe drinking water, inadequate sanitation and poor hygiene [6,33]. Other research at the micro level has shown that improved availability and use of piped water would have substantial effects on reducing urban mortality, especially during the post-neonatal and childhood periods [25]. Immunization on the other hand remains one of the most cost-effective health interventions likely to help achieve the MDG on reducing child mortality [5]. The WHO/UNICEF global immunization strategy for the years 2006–2015 [28] noted regrettably that immunization coverage has increased only marginally in some regions of the world since the early 1990s, and emphasizes the need to improve existing levels of vaccine coverage, particularly by accessing hard-to-reach populations on a regular basis.

Results from the Kenya case study indicate that urban under-five mortality has increased over time, and importantly, that urban poor children have higher mortality than even their rural counterparts. They also indicate that Kenyan poor women have almost three times more children than the rich, and are three times less likely to use contraceptives and three times more likely to have unmet need for family planning. Such poor reproductive health outcomes could further worsen child health outcomes among the poor. Another case study is that of Zambia, which has relatively high urbanization for the region. Periods of severe economic hardship in the early and mid 1990s were accompanied with increasing childhood mortality. This increase in mortality was observed among the urban population, particularly among the urban poor. In sub-Saharan Africa, the growing poverty and growing poor-rich fertility gap will result in greater proportions of children increasingly born to poorer families, with predictable implications for future population growth and the attainment of the health MDGs.

Rapid rate of urban population growth creates pressures on available infrastructure which, in many parts of Africa, have remained stagnant. The poor will increasingly lose out in such instances as the Kenya and Zambia case studies show. Consequently, the urban poor will experience many of the health challenges associated with lack of access to these basic amenities and services. The impact of this burden on the urban poor, combined with their growing size and proportion as many economies stagnate, creates an overall worsening of health indicators across urban areas in SSA [19]. Where evidence exists, huge inequities are observed among the poor and non-poor in urban areas of SSA [15,18,34].

Some of the findings of this study are rather counter-intuitive. For example, Tanzania and Malawi, and to a lesser extent, Madagascar, have higher rates of urban growth and poorer improvement in access to safe drinking water and child vaccination. Yet, they display strong declines in child mortality. By contrast, Côte d'Ivoire has increasing mortality despite noticeable increase in water supply and child vaccination. Country-specific policies and program context may explain some of these contrary-to-expectation results. With support from various development partners, Madagascar has implemented sound strategies of vitamin A supplementation, de-worming, insecticide-treated bed nets and oral rehydration salts, among others, to reduce child mortality and morbidity [35]; Malawi has put in place measures to address various child health and survival issues including developing nutrition rehabilitation strategies, providing insecticide-treated bed nets, and providing cholera treatment centers with essential drugs and other materials and training of health personnel on early detection and case management [36]; and the Tanzanian government has shown consistent commitment to invest in health and decentralize decision-making for health spending based on district priorities [37].

### Study limitations

The study has some limitations. All retrospective survey data in general, and birth history data in particular, are subject to biases arising from faulty respondent recall, the most common of which are completeness or displacement of birth dates; misreporting of age at death; deaths omission especially for infants who died early in life in the distant past; and survival bias since birth history data are limited to the experience of children born to surviving mothers [38]. Despite these limitations, several reports have indicated that the quality of DHS data to directly estimate infant and child mortality rates and to compare trends over time is generally good [38,39].

The paper presumes a constant rate of change in mortality, access to clean water and immunization coverage during the inter-survey period. This may not be the case in all countries. However, the constant rate approach has the advantage of summarizing in a single coefficient the change between the first and the last surveys. The study has other limitations including the exclusion of three or four outliers (out of 22 countries) in the correlation analysis, which represents a non-negligible proportion. For example, the strong association between trend in child vaccination and change in mortality (r = -0.73; p < 0.00) is substantially weakened if the three outliers (Côte d'Ivoire, Malawi and Uganda) are included in the analysis (r = -0.42; p < 0.10). The analysis does not take into account the standard errors of the estimates in the calculation of percentage change over time in each of the indicators. Since DHS surveys are based on population samples, some of the differences presented may not be statistically significant. Variance estimation of mortality rates requires the use of specialized software to perform complex calculations (i.e. Jacknife repeated replication methods), which was beyond the scope of the study.

Another limitation of the study is that identification of slum areas in DHS samples is not straightforward. In addition, urban samples are often small that trying to isolate intra-urban differences can sometimes be problematic. The estimates that we present of slum childhood mortality and vaccination rates are based on our definition of slums (absence of a flush toilet), which may not be fully accurate. The NCSS survey was a representative survey of slum households in Nairobi and our assertion of the disadvantage of slum children compared even with rural children, is strengthened by that survey's findings. We recommend that NCSS-type surveys should be undertaken periodically to provide good data for the study of intra-urban health differentials. Finally, the effects of HIV and AIDS are not included in the analysis, as previously indicated.

## Conclusion

Overall, the results of this analysis suggest that the urban poor should not be neglected in policy attention and resource allocation. While the poorest families and neighborhoods are the most likely to need interventions to prevent illnesses in children, existing evidence from SSA suggests that they are often the least likely to receive them, which not only adversely affects the health and survival of their children, but also pushes them further into indebtedness and poverty [33]. Failing to appropriately target the growing sub-group of the urban poor and improve their living conditions and health status – which is an MDG target itself – may result in lack of improvement on national indicators of health. This may consequently move countries further away from achieving the MDGs. In addition to improving the overall urban and national averages of health indicators, it is important to analyze, track and purposefully reduce health inequities – inequalities that are unjust and unfair, and ethically indefensible [16,40,41], since progress towards the achievement of the health MDGs will not automatically benefit the underprivileged population sub-groups [33,42]. The concern for equity therefore applies even to countries witnessing a substantial decline in child mortality. Progressive and sustained expansion of access to safe water supplies and vaccination coverage among disadvantaged urban dwellers, will contribute greatly to reducing under-five mortality from major causes of death in urban sub-Saharan Africa, and consequently, put countries on track to meeting the MDG target. The implementation of these interventions could be a measure of the attention paid by governments and development partners towards equity in the provision of health-related services.

## Competing interests

The authors declare that they have no competing interests. The study was supported with grants from the Wellcome Trust, the Hewlett Foundation and the Rockefeller Foundation. The sponsors had no involvement in the study design; in the collation, analysis, and interpretation of data; in the writing of the report; and in the decision to submit the paper for publication.

## Authors' contributions

JCF framed the research question, conducted the literature review, led in the data analysis, contributed in the writing of the paper. ACE conceived the idea of this manuscript and provided the overall guidance for the write up. NJM contributed in the data analysis and in the writing of the manuscript. JC was responsible for collating data for the study and also contributed in the data analysis. All authors read and approved the final manuscript.

## Pre-publication history

The pre-publication history for this paper can be accessed here:


